# Analysis of Metallo-β-lactamases, *oprD* Mutation, and Multidrug Resistance of β-lactam Antibiotic-Resistant Strains of *Pseudomonas aeruginosa* Isolated from Southern China

**DOI:** 10.1007/s00284-020-02148-3

**Published:** 2020-08-12

**Authors:** Fei Li, Danna Chen, Lijuan Li, Dezhi Liang, Fengping Wang, Bashan Zhang

**Affiliations:** Clinical Laboratory‚ Affiliated Dongguan People’s Hospital, Southern Medical University, No.3 Xinguchong Wandao South Road, Wangjiang District, Dongguan, 523059 Guangdong China

## Abstract

The purpose of this study was to analyze the metallo-β-lactamases (MBLs) genotype and *oprD* mutations of the β-lactam antibiotic-resistant *Pseudomonas aeruginosa* (PA) strains isolated from southern China. We collected 110 strains of β-lactam antibiotic-resistant PA from 2 hospitals during January 2016–December 2017 from Dongguan, South China. MBLs were detected, amplified, and typed using EDTA disc synergy test, PCR, and Sanger gene sequencing. The mutations and expression levels of *oprD* were detected using Sanger gene sequencing and qPCR. A total of 16.36% (18/110) β-lactam antibiotic-resistant PA strains produced MBLs, and the main genotypes of MBLs were IMP-25, VIM-2, and SIM-2. Sanger gene sequencing results showed that 107 of the 110 strains harbored mutations in *oprD* sequence, while 3 strains were negative for *oprD* amplification (2.73%). Among the 107 strains with positive amplification (97.27%), the rate of intentional mutations (including deletions, insertions, and premature stop codons) was 93.46% (100/107) and that of no disrupted mutation was 6.54% (7/107). qPCR analysis confirmed that the expression level of the OprD protein in the 7 strains of no disrupted mutation was significantly reduced. Among the β-lactam antibiotic-resistant PA strains in southern China, 16.36% were positive for MBLs. The loss rate of *oprD* was 2.73%, and almost all PA strains showed *oprD* amplification variation or transcription downregulation. Thus, impaired *oprD* expression and MBLs production may be some of the mechanisms of β-lactam antibiotic-resistance of PA strains in southern China.

**Electronic supplementary material:**

The online version of this article (10.1007/s00284-020-02148-3) contains supplementary material, which is available to authorized users.

## Introduction

*Pseudomonas aeruginosa* (PA) is a common opportunistic pathogen that can cause infections in immunocompromised patients or in patients with burns. Since the 1990s, the incidence of clinical isolates of PA has increased [1]. Clinically, β-lactam antibiotics are one of the most commonly used antibiotics for treatment of PA infections. However, the use of antibiotics for a long time may increase the drug resistance rate and multidrug resistance to β-lactam antibiotics in PA, thereby leading to difficulties in the treatment of PA infections [2, 3]. China’s monitoring report suggests that PA resistance to various antimicrobial agents is quite high in 2016 [4]. Therefore, the study of β-lactam antibiotic resistance and multidrug resistance mechanisms in PA is conducive to the prevention and treatment of PA infection.

Metallo-β-lactamases (MBLs), also known as class B β-lactamases, are carbapenemases that are usually identified in PA. MBLs can effectively hydrolyze β-lactams, including carbapenems, and aminoglycosides, and other antibiotics such as quinolones [5]. Various types of MBLs have been identified in PA, including IMP, VIM, SPM-1, GIM-1, SIM-1, NDM-1, and FIM-1. The production of MBLs is considered to be one of the important mechanisms for PA resistance [6, 7]. In addition, outer membrane porins, like OprD, that uptake amino acids, peptides, and carbapenems undergo insertion, frameshift, and mutation-mediated gene inactivation or expression suppression, which can increase carbapenem resistance in PA [8–11].

Although the production of MBLs and the inactivation of *oprD* in PA resistant to carbapenems have been reported in specific countries and regions such as Korea or in specific populations such as patients with fibrosis in South Korea [1, 9], the drug resistance mechanisms of β-lactam antibiotic-resistant strains of PA in China have not been still elucidated. The aim of this study was to analyze the drug susceptibility profiles of 110 isolates of β-lactam antibiotic-resistant PA from Dongguan, South China, and to determine the expression of MBLs and *oprD* for revealing the drug resistance mechanisms in β-lactam antibiotic-resistant PA. This study is expected to provide valuable information on the drug resistance in PA for the diagnosis and treatment of infections caused by PA.

## Materials and Methods

### Bacterial Isolates and Susceptibility Testing

*Pseudomonas aeruginosa* samples isolated from the patients who visited Dongguan People’s Hospital and Dongguan Donghua Hospital from 2016 to 2017 were stored in skimmed milk at − 70 °C. The strains were reconstituted from skimmed milk to a blood plate, and a single colony was picked from the blood plate. Antibiotic susceptibility and drug resistance of each strain was measured using the VITEK 2 Compact system (BioMérieux, Lyon, France). Drugs that were used for antibiotic susceptibility determination were ampicillin, ampicillin/sulbactam, piperacillin/tazobactam, cefazolin, cefotetan, ceftazidime, ceftriaxone, cefepime**,** imipenem, amikacin, gentamicin, tobramycin, ciprofloxacin, levofloxacin, nitrofurantoin, meropenem, and compound sulfamethoxazole. Multiple β-lactam antibiotic-resistant samples were selected for subsequent analyses.

### Pulsed-Field Gel Electrophoresis (PFGE)

A single colony was picked, inoculated on a nutrient plate, and incubated overnight at 37 °C. The concentration of the bacterial suspension was adjusted with a DENSIMAT turbidimeter with a turbidity of 3.8–4.2. Next, 1% Seakem Gold Glue was prepared. Bacterial genomic DNA was isolated on the gel block. The enzyme was digested with 40 U SpeI (Takara Bio, Otsu, Japan) endonuclease and incubated at 37 °C for 4 h. PFGE was performed in a CHEF-DRIII (Bio-Rad Laboratories, CA, USA) electrophoresis apparatus. The electrophoresis parameters were 5–15 s for 9 h and 15–50 s for 9 h. After the electrophoresis was completed, the gel was stained with a GelRed nucleic acid dye. Correlation analysis of PFGE patterns was performed using the BioNumerics software version 4.0 (Applied Maths, Sint-Martens-Latem, Belgium).

### Ethylenediaminetetraacetic Acid (EDTA) Disc Synergy Test

Since divalent cations (usually zinc) act as cofactors for maintaining the MBL enzyme activity and are inhibited by EDTA, EDTA is often used to detect MBL activity in PA [12]. Each isolate was inoculated in the Mueller–Hinton broth at 1 × 10^6^ CFU/mL and then inoculated on the agar medium with a cotton swab. Two discs (antibiotic disc and antibiotic + EDTA disc) were placed on the agar surface using sterile forceps with a spacing of approximately 30 mm. The EDTA disc was prepared by adding 250 μL of neutral EDTA to imipenem. The bacteriostatic inhibition zone was observed by incubation at 37 °C for 24 h. When the inhibition zone was > 4 mm in diameter, the strain was considered to be an MBL-positive phenotype. In addition, a control tray containing only EDTA was used to determine the activity of EDTA to ensure that the test isolate was inhibited by EDTA without causing false-positive results.

### *Mbls *and *oprD* Gene Amplification

DNA of each bacterial isolate was extracted using the TaKaRa Ex Taq® Hot Start Version (RR006Q, TaKaRa, Japan). Fragments of *mbls* and full-length *oprD* were amplified with the primers presented in Supplementary Table 1. The PCR program was set as follows: initial denaturation at 94 °C for 5 min; followed by 30 cycles of 94 °C for 30 s, 52 °C for 40 s, and 72 °C for 50 s; and final extension at 72 °C for 5 min. The PCR products were sequenced for gene typing using Sanger sequencing (Sangon Biotech, Shanghai, China).

### *OrpD* Mutation and Expression

Total RNA was isolated from the β-lactam antibiotic-resistant strains using the Trizol reagent (Thermo Fisher Scientific, IL, USA) according to the standard protocol. cDNA was synthesized using M-MLV Reverse Transcriptase (Promega, WI, USA). *OprD* was amplified using the Bestar qPCR RT Kit (DBI Bioscience, Ludwigshafen, Germany) according to the manufacturer’s instructions. The relative expression of *oprD* was normalized to that of the PAO1 standard strain and calculated by the 2^−ΔΔct^ method. *RpsL* was used as the internal control.

## Results

### Drug Sensitivity and Resistance of β-Lactam Antibiotic-Resistant PA

The 110 strains of PA isolated from Dongguan were resistant to β-lactams, fluoroquinolones, tetracyclines, and sulfonamides. Among them, 100%, 100%, 98.18%, 97.27%, and 96.36% of the isolates were resistant to imipenem, ampicillin, nitrofurantoin, compound sulfamethoxazole, and cefazolin, respectively. Approximately, 31.82%, 27.27%, and 26.36% of the strains were resistant to ceftazidime, ciprofloxacin, and levofloxacin, respectively. The resistance rate to amikacin was 10.00%, and the resistance rates to tobramycin, gentamicin, cefepime, and piperacillin/tazobactam were 17.27%, 19.09%, 21.82%, and 21.82%, respectively (Table 1).

**Table 1:** Sensitivity of β-lactam antibiotic-resistant *Pseudomonas aeruginosa* (PA) to commonly used antibiotics

Antibiotics	Sensitivity	Resistance
Imipenem	0 (0%)	110 (100%)
Ampicillin	0 (0)	110 (100%)
Nitrofurantoin	2 (1.82%)	108 (98.18%)
Compound sulfamethoxazole	3 (2.73%)	107 (97.27%)
Cefazolin	0 (0%)	106 (96.36%)
Cefotetan	2 (1.81%)	104 (94.55%)
Ampicillin/sulbactam	7 (6.36%)	103 (93.64%)
Ceftriaxone	6 (5.45%)	100 (90.91%)
Meropenem	14 (12.73%)	82 (74.54%)
Ceftazidime	54 (49.09%)	35 (31.82%)
Ciprofloxacin	64 (58.19%)	30 (27.27%)
Levofloxacin	69 (62.73%)	29 (26.36%)
Piperacillin/tazobactam	47 (42.73%)	24 (21.82%)
Cefepime	75 (68.18%)	24 (21.82%)
Gentamicin	78 (70.91%)	21 (19.09%)
Tobramycin	83 (75.45%)	19 (17.27%)
Amikacin	96 (87.27%)	11 (10%)

### MBLs Production in β-Lactam Antibiotic-Resistant PA

The EDTA disc synergy test showed that there were 18 (16%) isolates that were MBL-positive PA. Further typing results showed that 9 isolates were IMP-25 (8.18%), 8 isolates were VIM-2 (7.27%), and 4 isolates were SIM-2 (3.64%); in addition, in 3 isolates, both VIM-2 and SIM-2 were identified. The sequence alignment of these isolates in the GenBank was found to be identical to IMP-25 (EU352796), VIM-2 (AF191564), and SIM-2 (KT013203).

### *OprD* Variation, Phylogenetic Analysis, and Expression Analysis of β-Lactam Antibiotic-Resistant PA

**Fig. 1:**
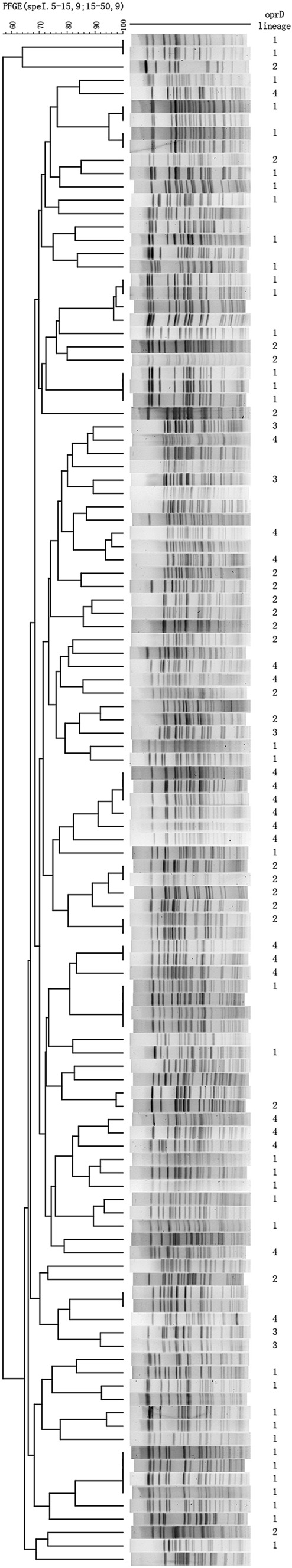
Pulsed-field gel electrophoresis (PFGE) profiles showing β-lactam antibiotic-resistant *Pseudomonas aeruginosa* (PA) isolates

The phylogenetic analysis of the 110 β-lactam antibiotic-resistant PA isolates according to the *oprD* sequence revealed that the gene could be divided into 4 classes, and each class had a sequence with high homology in GenBank. The standard strain (PAO1) reference sequence was in the third class (Fig. 1).

The *oprD* typing results showed that 107 isolates were positive for *oprD*, while 3 were negative. The rate of *oprD* deletion rate was 2.73%. Further sequencing of the *oprD*-positive isolates revealed that there were no disrupted mutations in 7 isolates, while there were frameshift mutations in 84 isolates and single-base single-point mutations in 16 isolates with premature stop codons (Table 2).

**Table 2:** Mutation of *oprD* in β-lactam antibiotic-resistant *Pseudomonas aeruginosa* (PA)

Type of mutation		Number of isolates	Percentage (%)
Frameshift	1-bp insertion	7	6.36
	More than 1-bp insertion	5	4.55
	1-bp deletion	8	7.27
	More than 1-bp deletion	56	50.91
	Insertion	8	7.27
Premature stop	Single mutation	16	14.55
Negative by PCR	Unknown large change	3	2.73
No disrupted mutation	Amino acid changes	7	6.36
Total		110	100

**Fig. 2:**
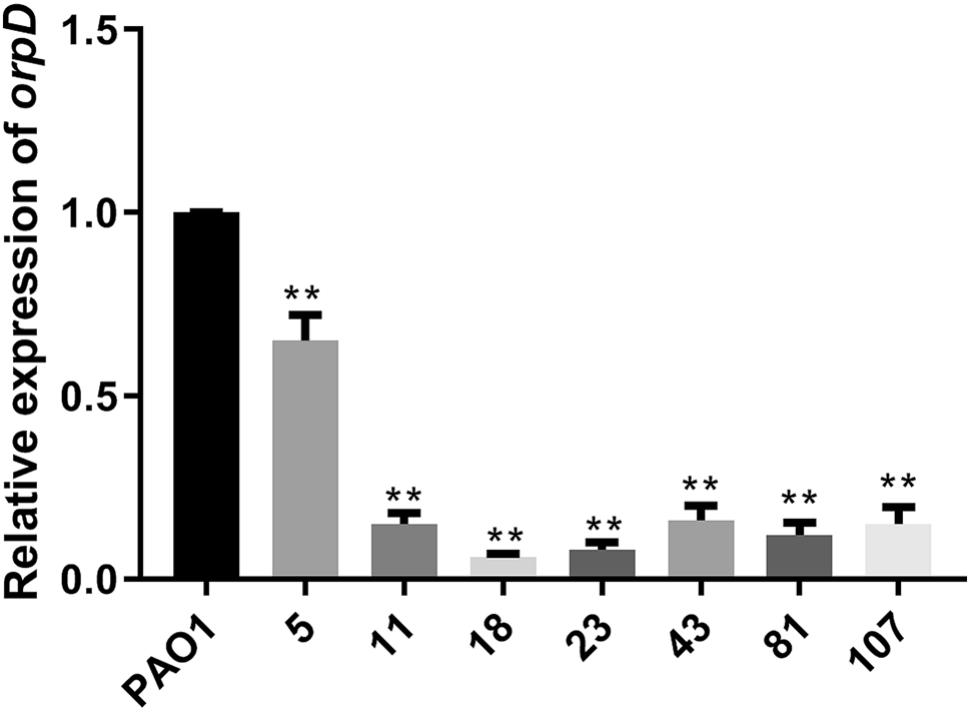
Relative expression of *oprD* expression in β-lactam-resistant *Pseudomonas aeruginosa* (PA) with no disruptive mutation

The mRNA expression levels of *oprD* were detected in the 7 non-disruptive mutant isolates (numbered: 5, 11, 18, 23, 43, 81, and 107). The qPCR results showed a significant decrease in the expression of *oprD* in all the isolates compared to that in the carbapenem-susceptible PAO1 strains (Fig. 2). In addition, we detected the expression of *oprD* in 10 imipenem-sensitive PA strains, however, did not find any strains with reduced *oprD* expression (data not shown).

## Discussion

There are only few studies on the drug resistance mechanisms of β-lactam antibiotic-resistant PA in southern China. One study had shown a mutation in *oprD* in imipenem-resistant PA in southern China [9]. Compared to the isolates in the previous study, the isolates in this study were characterized by multiple β-lactam antibiotic-resistance, and we analyzed the production and typing of MBLs from these isolates. We found that 18 of the 110 isolates of the β-lactam antibiotic-resistant PA in Dongguan, China, produced MBL, and 107 isolates were positive for *oprD* amplification. Further phylogenetic analysis of β-lactam antibiotic-resistant PA based on the *oprD* sequence revealed that these resistant isolates were mainly divided into 4 groups. *OprD* sequence analysis revealed a variation in *oprD* sequence of most β-lactam antibiotic-resistant PA strains. Among them, 84 isolates showed frameshift mutations, 16 showed premature stop codons, and 7 showed non-disruptive mutations. In particular, the 7 non-disruptive mutants showed a significant decrease in the gene expression level of *oprD*.

Currently, acquired MBLs include IMP, VIM, SPM, GIM, SIM, AIM, KHM, DIM, NDM, with IMP and VIM being the most common ones [13]. IMP, VIM, SPM, SIM, GIM, and NDM are of epidemiological and clinical significance in antibiotic resistance [14–16]. A previous national PA multidrug resistance test across 27 hospitals—mostly from southeastern China and southern China—shows that 8.2% of the strains produce MBLs, with IMP-9 being the most common one, followed by VIM-2 [17]. Another study shows that approximately 13 (35.1%) of the 37 multidrug-resistant PA strains isolated from the Hunan Province, China, produced IMP-4 and VIM-2 [18]. In our study, 18 resistant isolates (16.36%) carried MBLs—mainly IMP-25, VIM-2, and SIM-2. This result suggested that IMP-25, VIM-2, and SIM-2 played important roles in β-lactam antibiotic-resistance. In terms of the major epidemic types, our research suggested that the dominant type of MBLs in the southern region differed from the dominant types in the Hunan Province and the rest of the country. Simultaneously, there was also a consensus that the IMP type was dominant, followed by the VIM-2 type. This result was consistent with worldwide trends, indicating a potential association of IMP and VIM with PA multidrug resistance.

The inactivation of *oprD* is considered to be one of the mechanisms of PA resistance. Both sequence insertion and mutation can affect the activity of *oprD* [10, 19]. Our study found that *oprD* sequence of 107 isolates was mutated, while 3 isolates showed gene loss. In particular, the PCR results verified that the expression of *oprD* was decreased in the isolates with non-disruptive mutations, suggesting that the function of the *oprD* of these isolates was impaired. In addition, 3 isolates showed negative *oprD* amplification, suggesting the absence of *oprD*. Thus, we hypothesized that *oprD*, including positive or negative *oprD* amplification, might show abnormality in expression and function. The expression of *oprD* has been reported to be downregulated in multidrug-resistant PA in China [9, 18]. Our study further provided evidence that *oprD* expression was reduced in the non-disruptive mutant isolates. Studies have suggested that more than 50% of isolates have consistent characteristics of MBL and *oprD* [20]. Our study also showed a similar trend of both impaired MBLs production and mutated *oprD* sequence, which further supported this opinion.

## Conclusion

In summary, we analyzed the MBLs production of the β-lactam antibiotic-resistant PA strains in southern China and the variations in the *oprD* sequence, which were the 2 important mechanisms of PA resistance. Our results indicated that the drug resistance mechanism of β-lactam antibiotic-resistant PA in southern China might be attributed to the impaired *oprD* expression and MBLs production. Our study further provided evidence of the MBLs production and the variation in the *oprD* sequence as the clue for the treatment of infections caused by β-lactam multidrug-resistant PA in southern China.

## Electronic supplementary material

Below is the link to the electronic supplementary material.

Supplementary file1 (DOCX 16 kb)

## Data Availability

The datasets generated during and/or analyzed during the current study are available from the corresponding author on reasonable request.
