# Supplementary material for: Analysis of Metallo-β-lactamases, oprD Mutation, and Multidrug Resistance of β-lactam Antibiotic-Resistant Strains of Pseudomonas aeruginosa Isolated from Southern China
Source: Curr Microbiol. 2020 Aug 12;77(11):3264–9. doi: 10.1007/s00284-020-02148-3 (PMC7536146; doi:10.1007/s00284-020-02148-3)
Supplement: Supplementary file 1 — Supplementary file1 (DOCX 16 kb) [file 284_2020_2148_MOESM1_ESM.docx]

**Supplemental table 1.** Primers used in this study.

| Gene ID | Primer sequences (5′→3′) | Product length (bp) |
| --- | --- | --- |
| *imp* | F-CTCACATTTCCATAGCGACA | 429 |
|  | R-TTGAATCCTTTTACCGTCTGC |  |
| *vim* | F- ATGTTCAMRYTTTTGAGTAAGTT | 801 |
|  | R- CTACTCAACGACTGAGCGATT |  |
| *spm* | F-TGCTTTTCCTGCCCGTGTTCC | 270 |
|  | R-GAGCATGGGGTCCTCCTCGT |  |
| *sim* | F-TACAAGGGATTCGGCATCG | 570 |
|  | R- TAATGGCCTGTTCCCATGTG |  |
| *gim* | F- TCGACACACCTTGGTCTGAA | 477 |
|  | R-AACTTCCAACTTTGCCATGC |  |
| *ndm* | F- GGTTTGGCGATCTGGTTTTC | 621 |
|  | R- CGGAATGGCTCATCACGATC |  |
| *oprD* | F- CGCTTCGGAACCTCAACTATCGC | 1412 |
|  | R- TCACCGGTACCTACGCCCTT |  |
| *rpsL* | F-CTCGGCACTGCGTAAGGTAT | 138 |
|  | R-CCCGGAAGGTCCTTTACACG |  |
